# An elastic net regression model for predicting the risk of ICU admission and death for hospitalized patients with COVID-19

**DOI:** 10.1038/s41598-024-64776-0

**Published:** 2024-06-22

**Authors:** Wei Zou, Xiujuan Yao, Yizhen Chen, Xiaoqin Li, Jiandong Huang, Yong Zhang, Lin Yu, Baosong Xie

**Affiliations:** 1 Shengli Clinical Medical College of Fujian Medical University, Fuzhou, 350013 China; 2 Department of Pulmonary and Critical Care Medicine, Fujian Provincial Hospital, Fuzhou, 350004 China; 3 Chongqing Nanpeng Artificial Intelligence Technology Research Institute Co., Ltd., Chongqing, 401123 China

**Keywords:** COVID-19, Elastic net regression, Prediction models, Medical research, Experimental models of disease

## Abstract

This study aimed to develop and validate prediction models to estimate the risk of death and intensive care unit admission in COVID-19 inpatients. All RT-PCR-confirmed adult COVID-19 inpatients admitted to Fujian Provincial Hospital from October 2022 to April 2023 were considered. Elastic Net Regression was used to derive the risk prediction models. Potential risk factors were considered, which included demographic characteristics, clinical symptoms, comorbidities, laboratory results, treatment process, prognosis. A total of 1906 inpatients were included finally by inclusion/exclusion criteria and were divided into derivation and test cohorts in a ratio of 8:2, where 1526 (80%) samples were used to develop prediction models under a repeated cross-validation framework and the remaining 380 (20%) samples were used for performance evaluation. Overall performance, discrimination and calibration were evaluated in the validation set and test cohort and quantified by accuracy, scaled Brier score (SbrS), the area under the ROC curve (AUROC), and Spiegelhalter-Z statistics. The models performed well, with high levels of discrimination (AUROC_ICU_ [95%CI]: 0.858 [0.803,0.899]; AUROC_death_ [95%CI]: 0.906 [0.850,0.948]); and good calibrations (Spiegelhalter-Z_*ICU*_: − 0.821 (*p*-value: 0.412); Spiegelhalter-Z_*death*_: 0.173) in the test set. We developed and validated prediction models to help clinicians identify high risk patients for death and ICU admission after COVID-19 infection.

## Introduction

The coronavirus disease 2019 (COVID-19) pandemic has been a major cause of mortality worldwide and caused an unprecedented global health crisis^1,2^. A surge in the number of critically ill patients in intensive care units (ICUs) all over the world has placed a burden on ICUs^3^. Health care systems have been strongly affected and collapsed in most countries worldwide as the pandemic unfolded^4^. During the peak of the pandemic, rapid diagnosis and assessment of COVID-19 patients at risk for death and ICU admission are very crucial to managing potential capacity challenges. In this scenario, predictive models using readily available data are capable of identifying those at risk of severe disease effectively, greatly contributing to resource allocation and triage^5^.

Logistic regression models were most common among those prediction models in medicine^6^. Regression-based scoring systems has been used to assist with triage of patients with COVID-19^7–9^. These investigations collectively underscore the utility of regression-based scoring systems in assisting clinicians with timely intervention decisions, crucial for mitigating in-hospital mortality. However, they rely on logistic regression which is one of conventional methods and these static scores may not fully capture patient progression, necessitating a deeper understanding of how to tailor interventions based on individual patient conditions. In recent years, significant progress in predictive modeling, particularly through the application of machine learning (ML) methodologies such as elastic net, has offered an advantage over logistic regression on forecasting capabilities and was preferable for model fitting because it can potentially retain collinear variables^10,11^. Although prior studies have published prognostic models for COVID-19 patients, the majority of them either rely on logistic regression not an elastic net regression model or develop prediction models for death or ICU admission only on critically ill adult patients admitted to the ICU with no external validation or with a small sample size^11–16^.

To date, taking account of the paucity of validated prognostic models for death and ICU admission for COVID-19 patients with relatively large sample size, we are committed to developing and validating prediction models using elastic net regression to support resource allocation and disease management. We applied a cross-validation framework for model development and carefully evaluated the generalizability of the prediction models on an independent test cohort.

## Methods

### Study design and patient population

**Figure 1:**
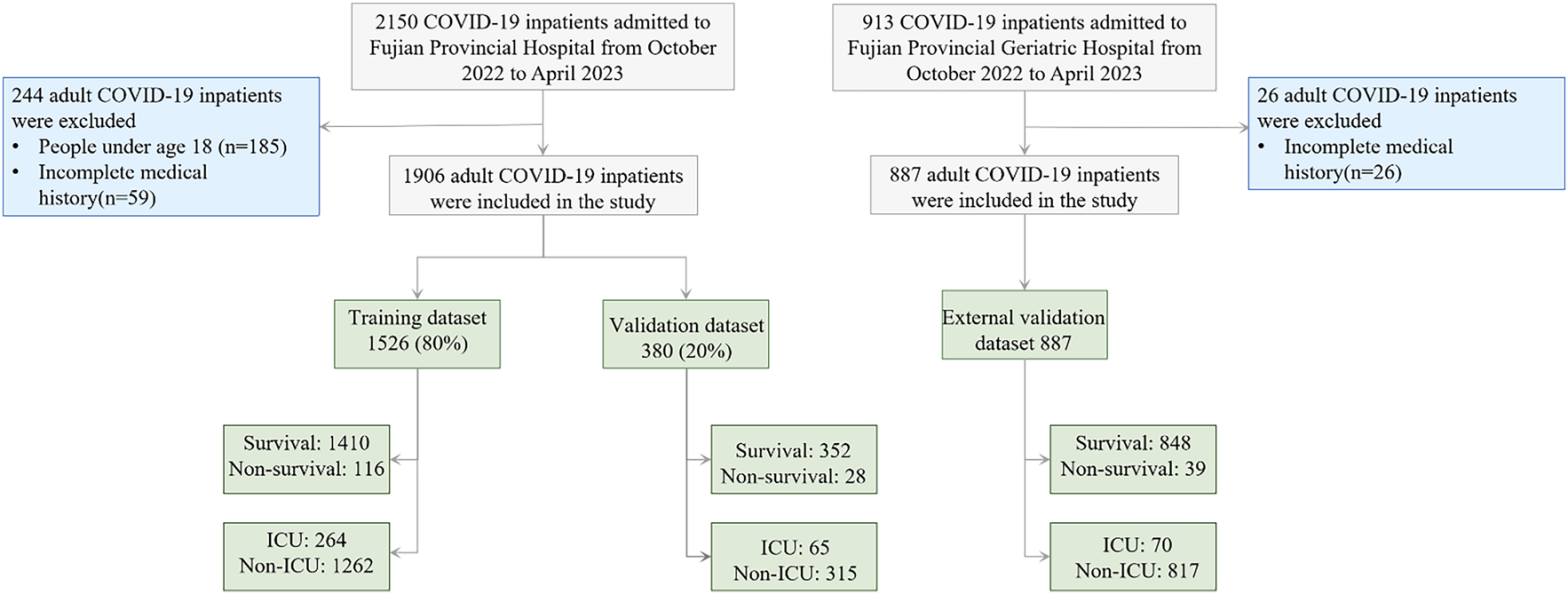
Flow diagram of the study. 1906 inpatients were included from Fujian Provincial Hospital finally by inclusion/exclusion criteria and inpatients were divided into derivation cohort (n = 1526) and test cohort (n = 380). 887 inpatients were included from Fujian Provincial Geriatric Hospital finally by inclusion/exclusion criteria for external validation.

The dataset includes demographic characteristics, clinical symptoms, comorbidities, laboratory results, treatment process and prognosis based on all adult (≥ 18 years of age) COVID-19 patients admitted to Fujian Provincial Hospital between October 2022 and April 2023. Patients with incomplete clinical and laboratory data were excluded. The diagnosis of COVID-19 is based on a positive reverse-transcriptase polymerase-chain reaction (RT-PCR) test. In this retrospective study, patients from the Fujian Provincial Hospital COVID-19 dataset were recruited split into training (n = 1526) and internal test (n = 380) data sets. Besides, external test cohort comes from patients from Fujian Provincial Geriatric Hospital (n = 887) (Fig. 1).

### Ethics approval and consent to participate

The studies involving human participants were reviewed and approved by the Ethics Review Committee of the Fujian Provincial Hospital (Grant No. K2023-12-016). All clinical investigations were conducted in accordance with the Declaration of Helsinki. We analyzed the data anonymously and written informed consent was waived by ethics committee of the Fujian Provincial Hospital due to the retrospective nature of our study of routine clinical data.

### Treatment protocols

Inpatient patients all received appropriate treatment based on their medical conditions. Treatment protocols of patients in the hospital include oxygen therapy, mechanical ventilation, nasal high flow oxygen therapy, invasive ventilation, thymus method, mask oxygen, prone position ventilation, nasal catheter for oxygen, extracorporeal membrane oxygenation (ECMO), antibiotic therapy, anticoagulant therapy glucocorticoid and antipyretic analgesics.

## Outcomes

The primary outcomes were in-hospital mortality and ICU admission. We developed and internally validated a prediction model for in-hospital mortality and ICU admission in the dataset form Fujian Provincial Hospital. Besides, we externally validated the prediction model in another dataset from Fujian Provincial Geriatric Hospital.

## Predictor variables

We extracted data from electronic medical records based on established risk factors and considered 161 candidate predictors, including demographic information (age, sex), clinical symptoms (cough, fever, fatigue, diarrhea, dry cough, etc.), comorbidities (hypertension, diabetes etc.) and treatments (oxygen therapy, mechanical ventilation, Extracorporeal membrane oxygenation, etc.). A full list of variables is available at Supplemental Materials Table S1.

## Statistical methods

### Preprocessing

The gathered data from 1906 patients were integrated and the duplicated records were removed. The original dataset consisted of 455 variables. We omitted variables that had more than 30% missing values to minimize bias resulting from missing data and finally a total of 161 variables remained. The missing values were imputed using the K-Nearest Neighbors (KNN) method to generate the analytical dataset.

### Model training

We employed elastic net, a regularized regression model^17^, on complete cases to develop and validate the risk prediction models. Samples were divided into two parts of a ratio of 8:2 using stratified sampling, where 80% of the total study samples were used for model development (derivation cohort). The remaining 20% of the samples were used as a test cohort for validation. The model was applied to an external dataset for external validation.

Risk prediction models were built using the derivation cohort. The derivation cohort was further divided into training and validation sets using a cross-validation framework^18^ (k = 3, repeats = 2) to allow interval validation. Specifically, the training set was randomly partitioned into three roughly equal-sized parts. We then left out one part as the validation set and the model was built on the remaining parts. The leave-out-modelling process was conducted recursively until each part was treated as a validation set for once. The cross-validation modelling process was repeated twice. Therefore, the number of training samples is double that of the original deviation cohort. We tuned one hundred combinations of hyperparameters, where we specified ten different alphas and ten different lambdas. For each combination of hyperparameters, six (k times number of repeats) prediction models were built for each outcome of interest. The model performance on the validation sets, which was calculated by averaging the model performance of the six models, was used to determine which combination of hyperparameters should be applied to our final prediction model. Finally, hyperparameters that generated the best model performance (highest value of scaled Brier score) were chosen and applied to the whole derivation cohort to obtain our final prediction model.

### Assessment of accuracy

We evaluated the model performance (overall performance, discrimination, and calibration) in the validation and test cohort. Accuracy, sensitivity, specificity, negative predictive values, positive predictive values and scaled Brier score were calculated to assess the overall performance of prediction models. The ROC curve was plotted and the area under the ROC curve (AUROC) was calculated to visualize and quantify the calibration^19^. Spiegelhalter-Z statistics^20^ were calculated to evaluate calibration. Calibration plots^21^ which compared the observed risks with the precited risks were provided to visualize the predictive ability of the risk prediction models. The effect coefficients of the included predictors in the risk prediction models.

## Results

### Participants

Overall, 1906 COVID-19 confirmed cases were included in our study. Of these, 1526 (event rate_*ICU*_: 17.3%, event rate_*death*_: 7.6%) inpatients were selected as the derivation cohort by stratified sampling and the remaining 380 (event rate_*ICU*_: 17.1%, event rate_*death*_: 7.4%) inpatients were used as test cohort. Table 1 presents the clinical characteristics of our study samples. In total, 329 (17.3%) patients were transferred to ICU. ICU inpatients were older (median: 76 years, interquartile range [IQR]: 65–83 years) than non-ICU inpatients (median: 72 years, IQR: 56–82 years), and the difference is statistically significant (*p*-value: 0.001). ICU inpatients more frequently underwent hypertension (62.6% versus 54.1%) and diabetes (42.2% versus 34.9%). Baseline characteristics for patients in our study who died vs survived are displayed in Table 2. 144 (7.6%) patients were non-survivors. The median age in non-survivors (median: 86 years, IQR: 80–91 years was significantly older than that of survivors (median:71 years, IQR: 56–81 years). The non-survivor group has higher proportions of patients with COVID-19 related symptoms including expectoration, cough, malaise fever, shortness of breath and fatigue. Baseline characteristics for patients in the development cohort (Fujian Provincial Hospital) and the external test cohort (Fujian Provincial Geriatric Hospital) are demonstrated in Table 3. The median age in the external test cohort (median: 85 years, IQR: 76–91 years) was significantly older than that in the development cohort (median: 73 years, IQR: 58–82 years). The external test cohort has higher proportions of patients with COVID-19 related symptoms including expectoration and cough than the development cohort (*p*-value: 0.001).

**Table 1:** Clinical and treatment characteristics of COVID-19 confirmed cases stratified by ICU admission status.

	ICU (N = 329) N (%)	Non-ICU (N = 1577) N (%)	*p*-value
Median age, years (IQR)	76.00 [65.00, 83.00]	72.00 [56.00, 82.00]	0.001
Sex			0.001
Male	225 (68.4)	925 (58.7)	
Female	104 (31.6)	652 (41.3)	
Death	53 (16.1)	91 (5.8)	< 0.001
Symptom(s)			
Expectoration	224 (68.1)	674 (42.7)	< 0.001
Cough	197 (59.9)	610 (38.7)	< 0.001
Malaise	167 (50.8)	481 (30.5)	< 0.001
Fever	151 (45.9)	476 (30.2)	< 0.001
Shortness of breath	155 (47.1)	378 (24.0)	< 0.001
Fatigue	68 (20.7)	289 (18.3)	0.361
Severity			
Mild	82 (24.9)	460 (29.2)	0.137
Moderate	25 (7.6)	310 (19.7)	< 0.001
Severe	52 (15.8)	200 (12.7)	0.152
Critical	57 (17.3)	70 (4.4)	< 0.001
Comorbidities			
Pneumonia	277 (84.2)	1023 (64.9)	< 0.001
Hypertension	206 (62.6)	853 (54.1)	0.006
Diabetes	139 (42.2)	551 (34.9)	0.014
Heart failure	131 (39.8)	435 (27.6)	< 0.001
Liver disease	71 (21.6)	408 (25.9)	0.118
Neoplastic disease	60 (18.2)	381 (24.2)	0.025
Arrhythmology	83 (25.2)	356 (22.6)	0.333
Stroke	65 (19.8)	306 (19.4)	0.944
Coronary heart disease	77 (23.4)	266 (16.9)	0.006
Abnormal liver function	80 (24.3)	238 (15.1)	< 0.001
Gastrointestinal hemorrhage	60 (18.2)	96 (6.1)	< 0.001
Treatment(s)			
Oxygen therapy	43 (13.1)	338 (21.4)	0.001
Mechanical ventilation	208 (63.2)	206 13.1)	< 0.001
Nasal high flow oxygen therapy	136 (41.3)	176 (11.2)	< 0.001
Invasive ventilation	179 (54.4)	87 (5.5)	< 0.001
Thymus method	114 (34.7)	145 (9.2)	< 0.001
Mask oxygen	54 (16.4)	181 (11.5)	0.017
Prone position ventilation	77 (23.4)	106 (6.7)	< 0.001
Nasal catheter for oxygen	44 (13.4)	37 (2.3)	< 0.001
ECMO	2 (0.6)	0 (0.0)	0.030
Antibiotic	298 (90.6)	1231 (78.1)	< 0.001
Antibacterial drug	294 (89.4)	1199 (76.0)	< 0.001
Anticoagulant therapy	280 (85.1)	1055 (66.9)	< 0.001
Glucocorticoid	203 (61.7)	714 (45.3)	< 0.001
Antipyretic analgesics	114 (34.7)	513 (32.5)	0.496

*ECMO* extracorporeal membrane oxygenation.

**Table 2:** Clinical and treatment characteristics of COVID-19 confirmed cases stratified by survival status.

	Non-survivors (N = 144) N (%)	Survivors (N = 1762) N (%)	*p*-value
Median age, years (IQR)	86.00 [80.00, 91.00]	71.00 [56.00, 81.00]	< 0.001
Sex	106 (73.6)	1044 (59.3)	0.001
Male			
Female			
ICU admission	53 (36.8)	276 (15.7)	< 0.001
COVID-19 symptom(s)			
Expectoration	90 (62.5)	808 (45.9)	< 0.001
Cough	72 (50.0)	735 (41.7)	0.065
Malaise	72 (50.0)	576 (32.7)	< 0.001
Fever	51 (35.4)	576 (32.7)	0.564
Shortness of breath	57 (39.6)	476 (27.0)	0.002
Fatigue	34 (23.6)	323 (18.3)	0.147
Severity			
Mild	21 (14.6)	521 (29.6)	< 0.001
Moderate	9 (6.2)	326 (18.5)	< 0.001
Severe	23 (16.0)	229 (13.0)	0.376
Critical	51 (35.4)	76 (4.3)	< 0.001
Comorbidities			
Pneumonia	137 (95.1)	1163 (66.0)	< 0.001
Hypertension	113 (78.5)	946 (53.7)	< 0.001
Diabetes	71 (49.3)	619 (35.1)	0.001
Heart failure	90 (62.5)	476 (27.0)	< 0.001
Liver disease	46 (31.9)	433 (24.6)	0.063
Neoplastic disease	45 (31.2)	396 (22.5)	0.022
Arrhythmology	71 (49.3)	368 (20.9)	< 0.001
Stroke	62 (43.1)	309 (17.5)	< 0.001
Coronary heart disease	61 (42.4)	282 (16.0)	< 0.001
Abnormal liver function	49 (34.0)	269 (15.3)	< 0.001
Gastrointestinal hemorrhage	28 (19.4)	128 (7.3)	< 0.001
Treatment(s)			
Oxygen therapy	134 (93.1)	1391 (78.9)	< 0.001
Mechanical ventilation	108 (75.0)	306 (17.4)	< 0.001
Nasal high flow oxygen therapy	69 (47.9)	243 (13.8)	< 0.001
Invasive ventilation	82 (56.9)	184 (10.4)	0.000
Thymus method	50 (34.7)	209 (11.9)	< 0.001
Mask oxygen	49 (34.0)	186 (10.6)	< 0.001
Prone position ventilation	28 (19.4)	155 (8.8)	< 0.001
Nasal catheter for oxygen	6 (4.2)	75 (4.3)	> 0.999
ECMO	1 (0.7)	1 (0.1)	0.145
Antibiotic	143 (99.3)	1386 (78.7)	< 0.001
Antibacterial drug	142 (98.6)	1351 (76.7)	< 0.001
Anticoagulant therapy	130 (90.3)	1205 (68.4)	< 0.001
Glucocorticoid	98 (68.1)	819 (46.5)	< 0.001
Antipyretic analgesics	81 (56.2)	546 (31.0)	< 0.001

*ECMO* extracorporeal membrane oxygenation.

**Table 3:** Clinical and treatment characteristics of COVID-19 confirmed cases between the development cohort and the external test cohort.

	Development cohort (N = 1906) N (%)	External test cohort (N = 887) N (%)	*p*-value
Median age, years (IQR)	73.00 [58.00, 82.00]	85.00 [76.00, 91.00]	< 0.001
Sex			0.026
Male	1150 (60.34)	495 (55.81)	
Female	756 (39.66)	392 (44.19)	
ICU admission	329 (17.26)	70 (7.89)	< 0.001
Death	144 (7.56)	39 (4.40)	0.002
COVID-19 symptom(s)			
Expectoration	898 (47.11)	506 (57.05)	< 0.001
Cough	807 (42.34)	490 (55.24)	< 0.001
Malaise	648 (34.00)	321 (36.00)	0.276
Fever	627 (32.90)	0 (0.00)	< 0.001
Shortness of breath	533 (27.96)	202 (22.77)	0.004
Fatigue	357 (18.73)	182 (20.52)	0.288
Severity			
Mild	542 (28.44)	0 (0.00)	< 0.001
Moderate	335 (17.58)	0 (0.00)	< 0.001
Severe	252 (13.22)	0 (0.00)	< 0.001
Critical	127 (6.66)	0 (0.00)	< 0.001
Comorbidities			
Pneumonia	1300 (68.21)	740 (83.43)	< 0.001
Hypertension	1059 (55.56)	631 (71.14)	< 0.001
Diabetes	690 (36.20)	398 (44.87)	< 0.001
Heart failure	566 (29.70)	421 (47.46)	< 0.001
Liver disease	479 (25.13)	353 (39.80)	< 0.001
Neoplastic disease	441 (23.14)	117 (13.19)	< 0.001
Arrhythmology	439 (23.03)	277 (31.23)	< 0.001
Stroke	371 (19.46)	461 (51.97)	< 0.001
Coronary heart disease	343 (18.00)	347 (39.12)	< 0.001
Abnormal liver function	318 (16.68)	200 (22.55)	< 0.001
Gastrointestinal hemorrhage	156 (8.18)	48 (5.41)	0.011
Treatment(s)			
Oxygen therapy	312 (16.4)	8 (0.90)	< 0.001
Mechanical ventilation	414 (21.72)	107 (12.06)	< 0.001
Nasal high flow oxygen therapy	312 (16.37)	8 (0.90)	< 0.001
Invasive ventilation	266 (13.96)	84 (9.47)	0.001
Thymus method	259 (13.59)	1 (0.11)	< 0.001
Mask oxygen	235 (12.33)	32 (3.61)	< 0.001
Prone position ventilation	183 (9.60)	315 (35.51)	< 0.001
Nasal catheter for oxygen	81 (4.25)	0 (0.00)	< 0.001
ECMO	2 (0.10)	0 (0.00)	1.000
Antibiotic	1529 (80.22)	9 (1.01)	< 0.001
Antibacterial drug	1493 (78.33)	4 (0.45)	< 0.001
Anticoagulant therapy	1335 (70.04)	0 (0.00)	< 0.001
Glucocorticoid	917 (48.11)	0 (0.00)	< 0.001
Antipyretic analgesics	627 (32.90)	3 (0.34)	< 0.001

*ECMO* extracorporeal membrane oxygenation.

### Model evaluation for COVID-19-associated mortality and ICU admission

**Figure 2:**
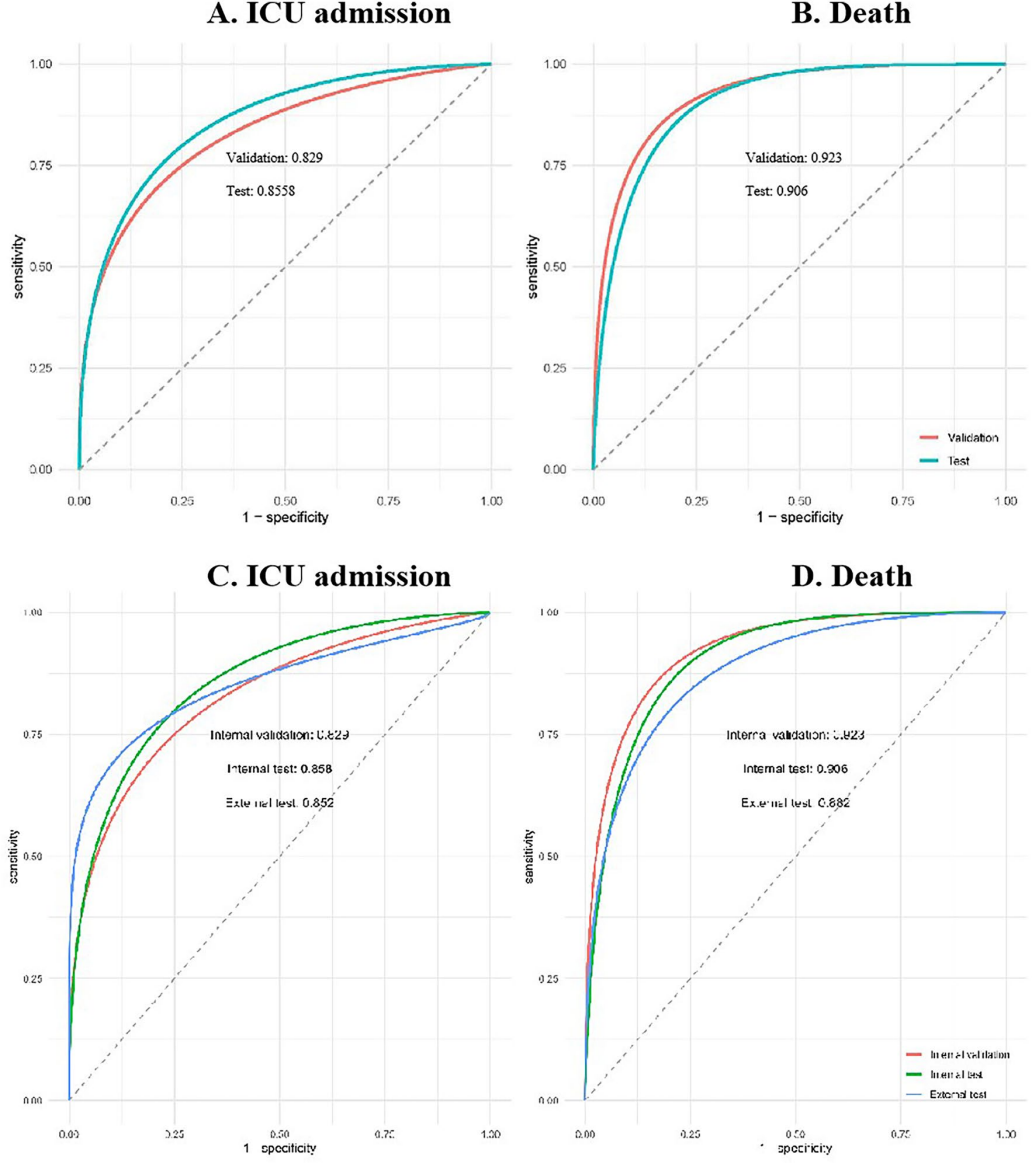
Risk prediction performance indices. risk prediction performance indices. (**A**) On the validation set and test cohort, the prediction model for ICU admission had an AUROC of 0.829 (95% CI: 0.810–0.850) and 0.858 (95% CI: 0.803–0.899) respectively. The value of test set is higher than validation set. (**B**) The model performance of risk prediction model for death performed similarly on the validation and test sets. AUROC reached above 0.900 on both validation set (AUROC: 0.923, 95% CI: 0.903–0.943) and test cohort (AUROC: 0.906, 95% CI: 0.850–0.948). The value of validation set is higher than test set. (**C**) The prediction model for ICU admission showed that AUROC was 0.829 (95% CI: 0.810–0.850) and 0.858 (95% CI: 0.803–0.899) on the validation set and test cohort. The value of test set is higher than validation set. (**D**) The prediction model for death showed that AUROC reached above 0.900 on both validation set (AUROC: 0.923, 95% CI: 0.903–0.943) and test cohort(AUROC: 0.906, 95% CI: 0.850–0.948). The value of validation set is higher than test set.

The performance of the final prediction models on the validation set and test cohort were demonstrated in Table 4 and Fig. 2. As was shown in Table 4, the prediction model for ICU admission shows good overall performance on the validation set and test cohort, with accuracy of 0.877 and 0.879 and scaled Brier score of 0.327 and 0.330, respectively. The Spiegelhalter-Z statistics with a value of − 0.285 (*p*-value: 0.776) on the validation set and − 0.821 (*p*-value: 0.412) on the test set indicate good calibration. ROC plots were provided to visualize the discrimination. AUROC was 0.829 (95% CI: 0.810–0.850) and 0.858 (95% CI: 0.803–0.899) on the validation set and test cohort (Fig. 2A), indicating good discrimination. In Table 5, we provide the sensitivity, specificity, positive predictive values (PPV) and negative predictive values (NPV) on the validation and test sets. We found that the prediction model for ICU admission and death achieved better sensitivity and PPV.

**Table 4:** Summary of model performance of prediction models on the validation and test sets.

Outcome	Data set	Acc	SbrS	AUROC (95% CI)	Spiegelhalter-Z (*p*-value)
ICU admission	Internal validation	0.877	0.327	0.829 (0.810,0.850)	− 0.285 (0.776)
	Internal test	0.879	0.330	0.858 (0.803,0.899)	− 0.821 (0.412)
	External test	0.943	0.352	0.852 (0.788, 0.907)	− 0.470 (< 0.001)
Death	Internal validation	0.935	0.328	0.923 (0.903,0.943)	− 0.818 (0.413)
	Internal test	0.932	0.256	0.906 (0.850,0.948)	0.173 (0.863)
	External test	0.953	0.169	0.882 (0.817, 0.931)	− 1.876 (0.061)

95% CI, 95% confidence interval; *Acc* accuracy; *SbrS* scaled brier score; *AUROC* area under the ROC curve.

**Table 5:** Sensitivity and specificity and negative and positive predictive values for prediction models on the validation and test sets.

Outcome	Data set	Sen	Spe	PPV	NPV
ICU admission	Internal validation	0.99 (0.98,0.99)	0.42 (0.31,0.54)	0.95 (0.94,0.97)	0.72 (0.58,0.87)
	Internal test	0.95 (0.94,0.96)	0.33 (0.27,0.38)	0.87 (0.85,0.89)	0.59 (0.51,0.67)
	External test	0.95 (0.92,0.97)	0.22 (0.11,0.31)	0.85 (0.81,0.89)	0.47 (0.28,0.66)
Death	Internal validation	0.99 (0.99,0.99)	0.29 (0.22,0.37)	0.94 (0.93,0.96)	0.72 (0.61,0.83)
	Internal test	0.99 (0.99,1)	0.31 (0.23,0.39)	0.95 (0.93,0.96)	0.73 (0.6,0.86)
	External test	0.98 (0.97,0.99)	0.29 (0.15,0.44)	0.97 (0.96,0.98)	0.42 (0.24,0.62)

*Sen* sensitivity; *Spe* specificity; *PPV* positive predictive value; *NPV* negative predictive value.

The model performance of risk prediction model for death performed similarly on the validation and test sets. The Accuracy was 0.935 for validation set and 0.932 for test cohort, SbrS of 0.328 for validation set and 0.256 for test cohort, respectively. AUROC reached above 0.900 on both validation set (AUROC: 0.923, 95% CI: 0.903–0.943) and test cohort (AUROC: 0.906, 95% CI: 0.850–0.948). The discrimination of risk prediction models for ICU admission and death was further assessed on an external dataset, with AUROC of 0.852 (95% CI: [0.788–0.907]) and 0.882 (95% CI: [0.817–0.931]), respectively.

**Figure 3:**
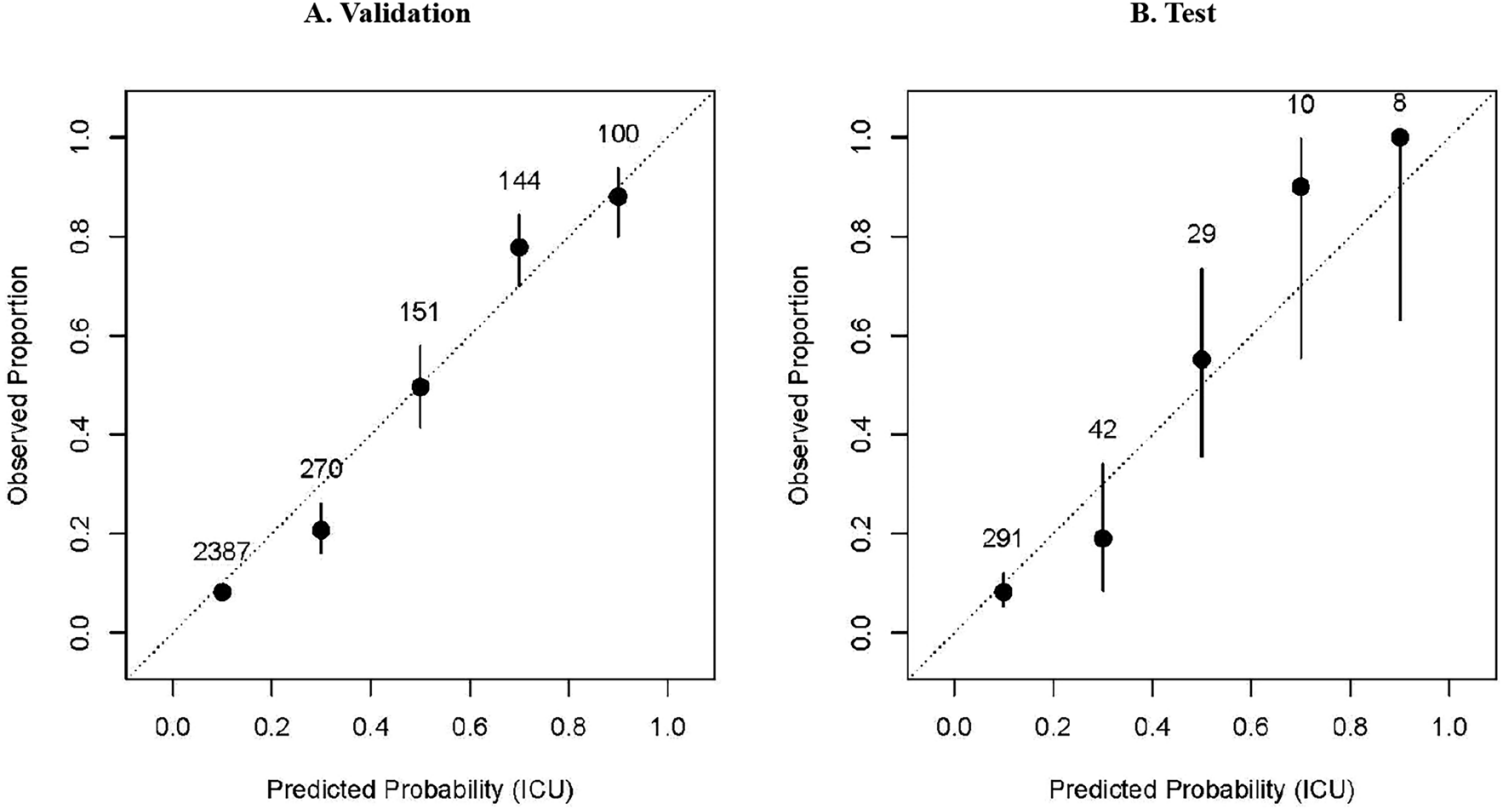
Calibration indices of risk prediction model of ICU admission. (**A**) The calibration of risk prediction model for ICU admission on the validation set. (**B**) The calibration of ICU admission risk prediction model on the test cohort. Figure 3 indicate that the model tends to overestimate the probability of ICU. In panel A, for instance, when the model predicts more than 0.3 probability of ICU, the observed proportion of ICU is closer to 0.2. This discrepancy suggests that the model is too progressive in its ICU estimates.

**Figure 4:**
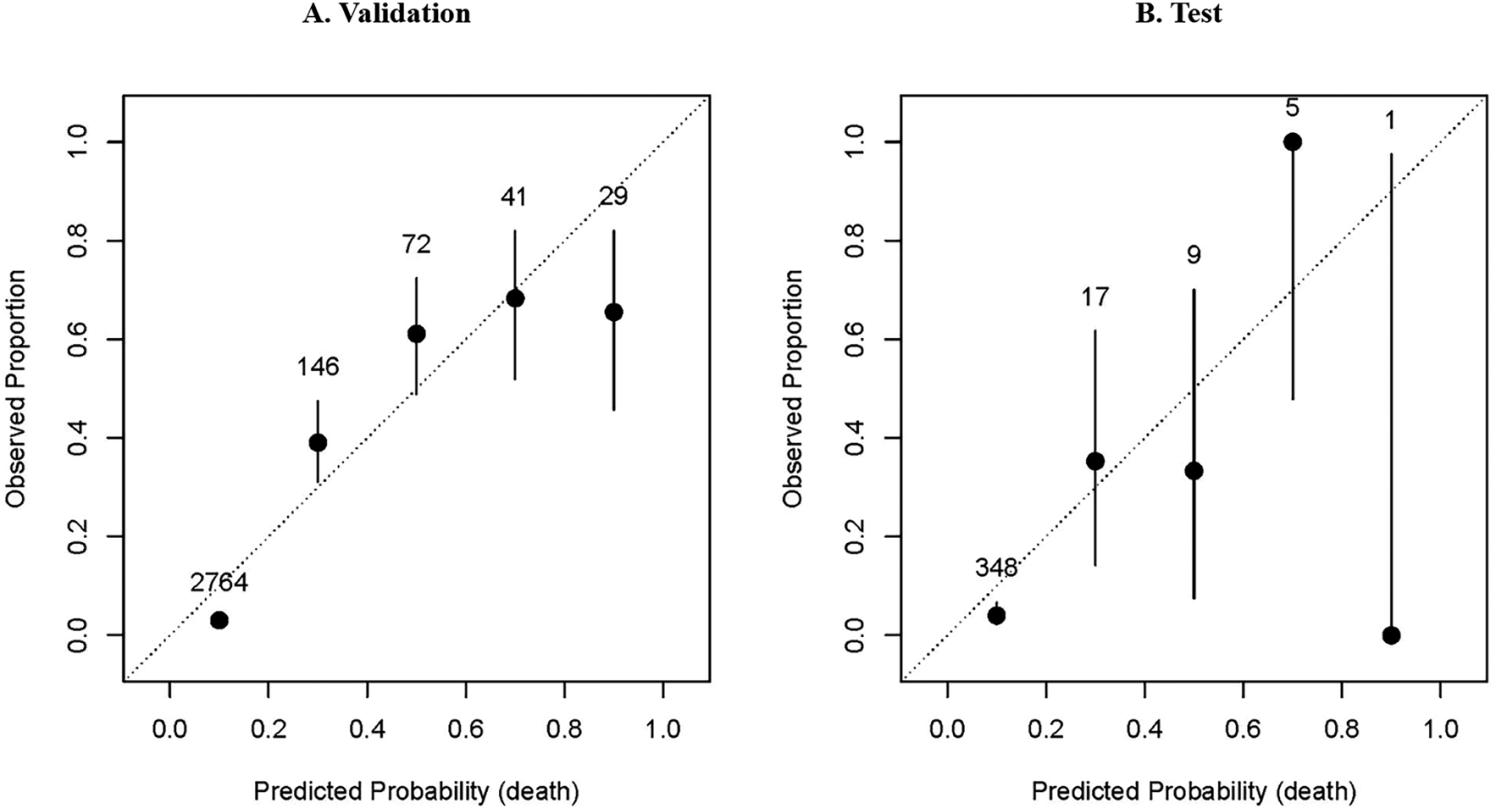
Calibration indices of risk prediction model of death. (**A**) Calibration indices of risk prediction model of death on the validation set. (**B**) Calibration indices of risk prediction model of death on the test cohort. Both panels in Fig. 4 indicate that the model tends to underestimate the probability of death. In panel A, for instance, when the model predicts a 0.9 probability of death, the observed proportion of death is less than 0.7. This discrepancy suggests that the model is too conservative in its death estimates.

The calibration plots were demonstrated in Figs. 3 and 4. The X-axis represents the model prediction probability value, and the Y-axis represents the actual observation proportion. By dividing the predicted value into several intervals, we calculated the average prediction probability and the true positive rate for each interval^22^. In this study, we divided the predicted values into five intervals, which correspond to five levels of risk categories (low-risk, medium–low, medium, medium–high, and high). If the values of Pred and True are close, then the coordinates of (Pred, True) will be close to the diagonal. Therefore, the closer each point is to the diagonal, the better the calibration ability of the model.

The calibration of risk prediction model for ICU admission on the validation set was showed in Fig. 3A. The data pairs (Pred, True) in the calibration plot for ICU admission were close to the diagonal line, indicating good predictive ability of the prediction model in the different risk groups. The calibration of ICU admission risk prediction model on the test cohort was showed in Fig. 3B. The predicted probabilities and observed risks were close in medium and low-risk subgroups (refers to samples with predicted risks lower than 0.6). However, the predicted probability in the high-risk groups was lower than the observed risks, indicating underestimation of ICU admission risk in high-risk groups.

The calibration of the risk prediction model for death on the validation set and test cohort was presented in Fig. 4. In the high-risk subgroup, the predicted probability was greater than the observed risk, indicating an overestimate of mortality risk. The data pairs in the rest of the subgroups were close to the diagonal line, indicating great calibration. However, we observed poor calibration on the test cohort, with overestimate of death risk in medium and high-risk subgroups and underestimate of death risk in medium–high risk subgroup.

**Figure 5:**
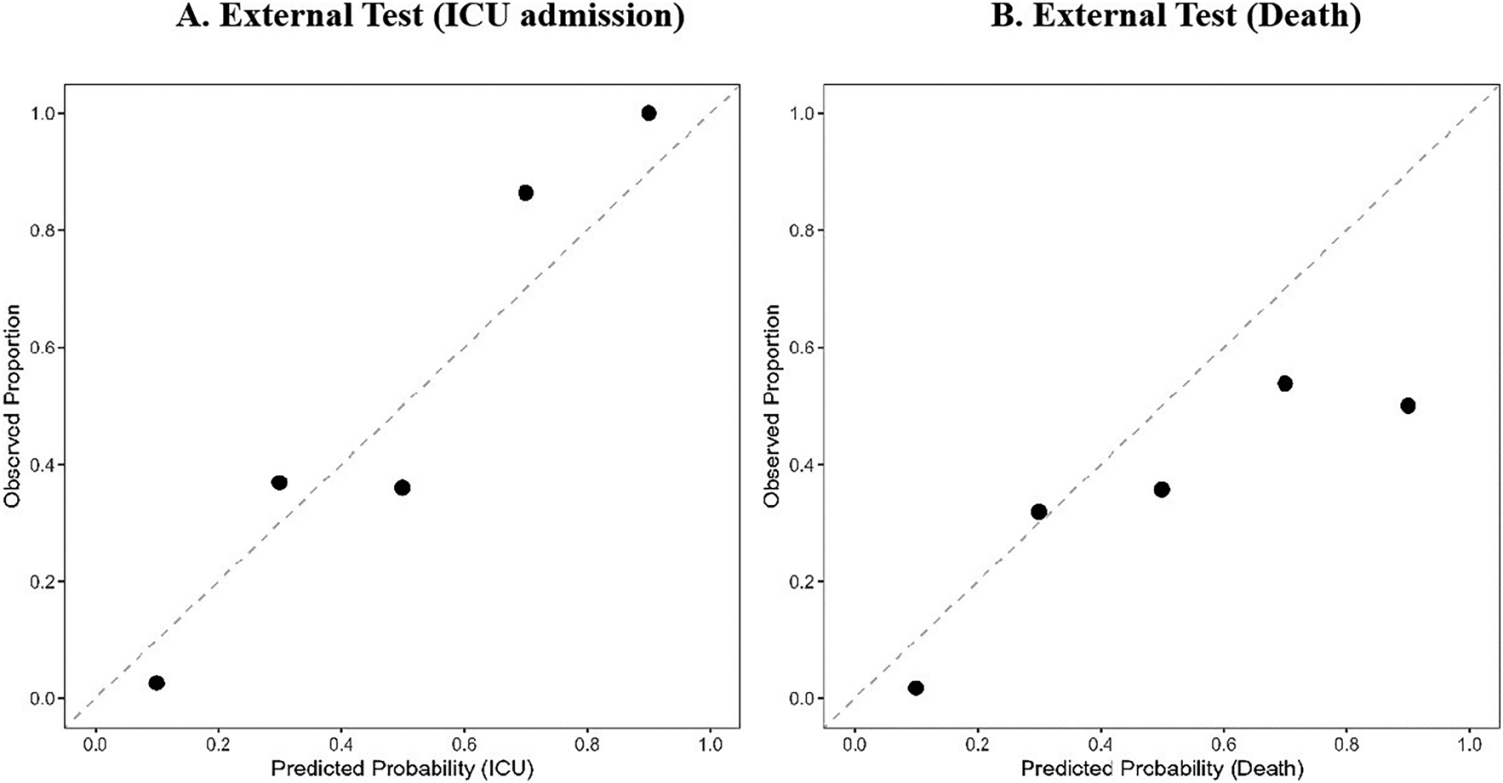
Calibration indices of risk prediction model of ICU admission /death on external dataset. (**A**) Calibration indices of the risk model for ICU admission. (**B**) Calibration indices of the risk model for death. Figure 5 indicates that the model overestimated the probability of death and underestimated the probability of ICU in external validation. For example, in panel A, when the model predicts an ICU probability less than 0.8, the observed ICU probability is higher than 0.8. In panel B, when the model predicts a mortality probability close to 0.8, the observed mortality probability is close to 0.6.

The calibration of the risk prediction models on the external dataset was showed in Fig. 5. We observed good calibration of the risk model for ICU admission (Fig. 5A). However, the risk prediction model for death overestimated risk for high-risk groups (Fig. 5B), indicating poor calibration.

## Discussion

In this study, we developed and validated risk prediction models for ICU admission and death for COVID-19 inpatients using Elastic Net model. Our study showed that the prediction models had the high discrimination and best calibration of the risk prediction model for ICU admission and death. It could identify patients at high or low risk of ICU admission and mortality. Due to the ongoing impact of the COVID-19 disease on health care systems, no wonder that there are prior studies about prognostic models for COVID-19 patients. In what follows, we outline the main characteristics of the most important studies and compare these works with ours in order to highlight the respective strengths and limitations.

Rahmatinejad et al.^7^ compared the performance of a variety of regression-based scoring systems, and highlighted the superiority of the APACHE II scoring system in predicting inhospital mortality of patients in the ICU, which was more suitable for critically ill adult patients admitted to the ICU directly from the ED (emergency departments). What sets it apart is the population included in our study, not only the more severe COVID-19 inpatients like in other studies^12,23,24^ but also all COVID-19 inpatients in our hospital. This broad inclusion criterion makes our study less prone to selection bias and improves the generalizability of our findings, as our study has confirmed that our model had higher discrimination in the external dataset demonstrating the generalizability and robustness of the model.

Rahmatinejad et al.^8^ compared the prognostic accuracy of six different regression-based scoring systems for predicting in-hospital mortality among Covid-19 patients with AUC values ranging between 70 and 80% and revealed the WPS, REMS, and NEWS have a fair discriminatory performance, which was reported relatively low performance compared with our study. In our study, the model achieved AUROC of 0.852 (95% CI: [0.788–0.907]) and 0.882 (95% CI: [0.817–0.931]) for ICU admission and death respectively, which were classified as a good performance on an external dataset comparable to or better than alternative COVID-19 prediction models^3,12,23,25–31^. Our models showed that a reliable prediction can be made for ICU admission and death in COVID-19 inpatients. We note that the authors report that multiple imputations within bootstrap samples were used. Thus, the reported results may be impacted by an over-estimation.

Rahmatinejad et al.^13^ developed logistic regression and ensemble learning models with the highest AUROC of 83.9% for predicting in-hospital mortality in emergency departments. While the reported results are comparable with our own, they validated the model only on the test dataset and limited themselves to three levels of ESI acuity, making it prone to overfitting and unclear as to what extent these models can be generalized to a broader ED population.

Famiglini et al.^27^ introduced a robust and parsimonious machine learning method to predict ICU admission in COVID-19 patients but the generalizability of the developed models was not externally validated. The difference is that our prediction models were applied to an external dataset and had higher discrimination in the external dataset, demonstrating the generalizability and robustness of our model.

Regarding ICU admission, Sabetian et al.^11^ reported that machine learning offered an advantage over logistic regression for predicting patients requiring intensive care. However, this study was based on a small sample of 506 patients.

It was encouraging to note that we incorporated four categories of clinical characteristics, symptoms, comorbidities and treatment information based on all patients’ data in our prediction models when making determinations related to ICU admission and death for COVID-19 inpatients. Our model finally identified 162 variables as being predictive of ICU admission and death, having more variables than other studies^23,31^. Some variables in our study have been reported as strong contributors to COVID mortality and ICU admission prediction in other studies^32,33^.Our prediction models were derived using these variables and were exported to an external dataset.

Since clinical judgment is subjective, models can be helpful to physicians, especially those who are not adequately experienced. Providing these models to physicians can help them better estimate patients’ prognoses. Rahmatinejad et al.^9^ revealed that regression-based mSOFA predicted a better-calibrated mortality risk than emergency residents’ judgment. Models can augment the physician’s clinical judgment. Elastic net regression is interpretable and can provide better prediction accuracy compared to standard statistical analysis^3,10,31^. As proved in this study, applying elastic net regression indeed achieved a good performance. It is noteworthy that we conducted risk stratification to five levels of risk categories. However, the calibration of the risk prediction model indicated underestimation of ICU admission risk in high-risk groups and death risk in medium–high risk subgroup and an overestimate of death risk in medium and high-risk subgroups. The possible reasons for the above situations were as follows: 1) Model Complexity: we employed elastic net, a regularized regression model in our study, which has a certain degree of complexity. Complex models may overfit the training data, leading to poor generalization and calibration performance on new data. 2) Data Quality: there are inconsistencies and biases in the data between the derivation cohort and the external test cohort, which can adversely affect model calibration.3) Missing Variables: the elastic net models in our study cannot adequately capture all relevant variables that influence the outcomes. Missing variables or unaccounted-for confounding factors can introduce bias and lead to calibration discrepancies.

This study has several strengths. First, we conducted this study with a larger sample size relative to certain studies to develop elastic net regression models for not only mortality but also ICU admission in all COVID-19 patients. Second, we presented external validation results across patients admitted to Fujian Provincial Geriatric Hospital. Although the performance degraded with external validation as opposed to internal validation, we could estimate the expected model performance by this approach when used on new patients from the same or other centers.

There are also several limitations to this work. First, we left out the test cohort as an artificial “external data” to evaluate the generalizability of our prediction models. However, the prediction models are prone to overestimate the performance since the test and derivation cohorts are of the same source. Our perdition model could be used for new patients whose characteristics are similar to those of our study samples. However, when applying it to new data with different features, the prediction results could be inaccurate. Second, this study was conducted during the COVID-19 pandemic, so our findings may vary in non-pandemic conditions. Third, we could only analyze those parameters that had been recorded and documented in our hospital's clinical electronic record system because this was a retrospective study. Various parameters, such as specific leukocyte/lymphocyte subgroups, tumor necrosis factor or interleukins, may be overlooked although they might prove to be better predictors than the parameters included in this study and might carry additional independent information. Fourth, considering that imputing missing values within each bootstrap sample can introduce additional variability into the analysis and increase the risk of model overfitting, these variables were removed from the analysis in our study. However, excluding variables with high missingness can introduce bias and limit generalizability.

In future works, we aim to further investigate our models by refining model variables, improving calibration, or validating the models in diverse populations and combine our methods with models predicting the worsening of health status over time^34^, thus providing clinicians with more information about patients’ health status and better risk stratification indications. Furthermore, it is necessary to conduct multicentric studies to further investigate the performance of these models in the COVID-19 inpatients.

In conclusion, the prediction models in this study can predict the risk of ICU admission and death for COVID-19 inpatients with good performance on an external dataset. COVID-19 ICU admission and death risk stratification tools can help physicians in patient stratification during the stages of the pandemic, ultimately improving care, informing accurate and rapid triage decisions and facilitating better resource allocation.

## Supplementary Information

Supplementary Information.

## Data Availability

The datasets analyzed during the current study are available from the corresponding author on reasonable request.
