# Supplementary material for: An elastic net regression model for predicting the risk of ICU admission and death for hospitalized patients with COVID-19
Source: Sci Rep. 2024 Jun 22;14:14404. doi: 10.1038/s41598-024-64776-0 (PMC11193779; doi:10.1038/s41598-024-64776-0)
Supplement: Supplementary file 1 — Supplementary Information. [file 41598_2024_64776_MOESM1_ESM.docx]

**ONLINE SUPPLEMENTARY MATERIALS**

**Title:** An Elastic Net Regression Model for predicting the risk of ICU admission and death for hospitalized patients with COVID-19

**Authors:**Wei Zou^1,2^**^†^**, Xiujuan Yao^1,2^**^†^**, Yizhen Chen^1^, Xiaoqin Li^1,2^, Jiandong Huang^1^, Yong Zhang^3^, Lin Yu^3^, Baosong Xie ^2^*****

Table S1. Clinical and treatment characteristics of COVID-19 confirmed cases stratified by survival status on internal and external dataset

|  | Internal data | | External data | |
| --- | --- | --- | --- | --- |
|  | **Survivors** | **Non-survivors** | **Survivors** | **Non-survivors** |
| n | 1762 | 144 | 849 | 38 |
| **Age (median [IQR])** | 71.00 [56.00, 81.00] | 86.00 [80.00, 91.00] | 85.00 [76.00, 90.00] | 90.50 [80.50, 94.00] |
| **Sex** |  |  |  | 23 (60.5) |
| **Male** | 1044 (59.3) | 106 (73.6) | 473 (55.7) |  |
| **Female** | 718 (40.7) | 38 (26.4) | 376 (44.3) | 15 (39.5) |
| **Increased lung texture** | 368 (20.9) | 15 (10.4) | 414 (48.8) | 10 (26.3) |
| **Multiple small spots** | 524 (29.7) | 26 (18.1) | 191 (22.5) | 6 (15.8) |
| **Pneumonia** | 1163 (66.0) | 137 (95.1) | 703 (82.8) | 37 (97.4) |
| **Respiratory failure** | 37 (2.1) | 48 (33.3) | 33 (3.9) | 22 (57.9) |
| **Asthma** | 37 (2.1) | 48 (33.3) | 33 (3.9) | 22 (57.9) |
| **Cerebral apoplexy** | 309 (17.5) | 62 (43.1) | 441 (51.9) | 20 (52.6) |
| **Coronary heart disease** | 282 (16.0) | 61 (42.4) | 329 (38.8) | 18 (47.4) |
| **arrhythmology** | 368 (20.9) | 71 (49.3) | 260 (30.6) | 17 (44.7) |
| **Myocardial infarction** | 70 (4.0) | 27 (18.8) | 35 (4.1) | 3 (7.9) |
| **Hypertension** | 946 (53.7) | 113 (78.5) | 603 (71.0) | 28 (73.7) |
| **Hyperkalemia** | 97 (5.5) | 33 (22.9) | 22 (2.6) | 2 (5.3) |
| **Metabolic acidosis** | 100 (5.7) | 45 (31.2) | 17 (2.0) | 7 (18.4) |
| **Septic shock** | 61 (3.5) | 42 (29.2) | 5 (0.6) | 4 (10.5) |
| **Blood coagulation dysfunction** | 108 (6.1) | 38 (26.4) | 110 (13.0) | 12 (31.6) |
| **Nasal catheter for oxygen** | 75 (4.3) | 6 (4.2) | 0(0.0) | 0(0.0) |
| **Mask oxygen** | 186 (10.6) | 49 (34.0) | 29 (3.4) | 3 (7.9) |
| **Thymus method new** | 209 (11.9) | 50 (34.7) | 1 (0.1) | 0 (0.0) |
| **Mechanical ventilation** | 306 (17.4) | 108 (75.0) | 85 (10.0) | 22 (57.9) |
| **Invasive ventilation** | 184 (10.4) | 82 (56.9) | 64 (7.5) | 20 (52.6) |
| **Noninvasive ventilation** | 265 (15.0) | 88 (61.1) | 85 (10.0) | 22 (57.9) |
| **Plasmapheresis** | 1 (0.1) | 0 (0.0) | 0(0.0) | 0(0.0) |
| **Antipyretic and analgesic** | 546 (31.0) | 81 (56.2) | 2 (0.2) | 1 (2.6) |
| **Gamma globulin (IVIG) for intravenous injection** | 106 (6.0) | 30 (20.8) | 1 (0.1) | 0 (0.0) |

Table S2. Clinical and treatment characteristics of COVID-19 confirmed cases stratified by ICU admission status on internal and external dataset

|  | **Internal data** | | **External data** | |
| --- | --- | --- | --- | --- |
|  | **Non-ICU** | **ICU** | **Non-ICU** | **ICU** |
| n | 1577 | 329 | 818 | 69 |
| **Age (median [IQR])** | 72.00 [56.00, 82.00] | 76.00 [65.00, 83.00] | 85.00 [76.00, 91.00] | 87.00 [82.00, 92.00] |
| **Sex** |  |  |  |  |
| **Male** | 925 (58.7) | 225 (68.4) | 457 (55.9) | 39 (56.5) |
| **Female** | 652(41.3) | 104 (31.6) | 361 (44.1) | 30 (43.5) |
| **Nasal obstruction** | 24 (1.5) | 4 (1.2) | 3 (0.4) | 1 (1.4) |
| **Fever** | 476 (30.2) | 151 (45.9) | 0(0.0) | 0(0.0) |
| **Hemoptysis** | 12 (0.8) | 3 (0.9) | 0(0.0) | 0(0.0) |
| **Dyspnea** | 46 (2.9) | 7 (2.1) | 10 (1.2) | 0 (0.0) |
| **Myodynia** | 40 (2.5) | 10 (3.0) | 33 (4.0) | 0 (0.0) |
| **Drowsiness** | 50 (3.2) | 17 (5.2) | 16 (2.0) | 6 (8.7) |
| **Headache** | 65 (4.1) | 14 (4.3) | 30 (3.7) | 5 (7.2) |
| **Dyspnea** | 378 (24.0) | 155 (47.1) | 167 (20.4) | 35 (50.7) |
| **Cough** | 610 (38.7) | 197 (59.9) | 449 (54.9) | 41 (59.4) |
| **Expectoration** | 674 (42.7) | 224 (68.1) | 459 (56.1) | 47 (68.1) |
| **Thoracodynia** | 86 (5.5) | 10 (3.0) | 0(0.0) | 0(0.0) |
| **Giddy** | 122 (7.7) | 10 (3.0) | 298 (36.4) | 25 (36.2) |
| **Poor mental response** | 481 (30.5) | 167 (50.8) | 295 (36.1) | 25 (36.2) |
| **Groan** | 2 (0.1) | 0 (0.0) | 0(0.0) | 0(0.0) |
| **Low-grade fever** | 204 (12.9) | 77 (23.4) | 101 (12.3) | 8 (11.6) |
| **Rash** | 11 (0.7) | 3 (0.9) | 5 (0.6) | 0 (0.0) |
| **Disturbance of consciousness** | 119 (7.5) | 51 (15.5) | 35 (4.3) | 16 (23.2) |
| **Tubercular shadow** | 30 (1.9) | 3 (0.9) | 41 (5.0) | 4 (5.8) |
| **Funiculus shadow** | 8 (0.5) | 0 (0.0) | 70 (8.6) | 2 (2.9) |
| **Lung consolidation** | 40 (2.5) | 10 (3.0) | 13 (1.6) | 0 (0.0) |
| **Grid shadow** | 95 (6.0) | 33 (10.0) | 15 (1.8) | 1 (1.4) |
| **Multiple ground glass shadows** | 97 (6.2) | 26 (7.9) | 128 (15.6) | 12 (17.4) |
| **Cardiac shadow enlargement** | 15 (1.0) | 7 (2.1) | 92 (11.2) | 8 (11.6) |
| **dermatomyositis** | 3 (0.2) | 0 (0.0) | 0(0.0) | 0(0.0) |
| **Myocarditis** | 10 (0.6) | 2 (0.6) | 2 (0.2) | 0 (0.0) |
| **Liver disease** | 408 (25.9) | 71 (21.6) | 300 (36.7) | 53 (76.8) |
| **Pneumonia** | 1023 (64.9) | 277 (84.2) | 674 (82.4) | 66 (95.7) |
| **Chronic obstructive pulmonary disease** | 42 (2.7) | 12 (3.6) | 49 (6.0) | 3 (4.3) |
| **Viral pneumonia** | 1543 (97.8) | 324 (98.5) | 0(0.0) | 0(0.0) |
| **Atypical interstitial pneumonia** | 7 (0.4) | 0 (0.0) | 11 (1.3) | 0 (0.0) |
| **Respiratory failure** | 48 (3.0) | 37 (11.2) | 29 (3.5) | 26 (37.7) |
| **Asthma** | 48 (3.0) | 37 (11.2) | 29 (3.5) | 26 (37.7) |
| **Cytomegalovirus pneumonia** | 2 (0.1) | 0 (0.0) | 0(0.0) | 0(0.0) |
| **Cerebral apoplexy** | 306 (19.4) | 65 (19.8) | 433 (52.9) | 28 (40.6) |
| **Coronary heart disease** | 266 (16.9) | 77 (23.4) | 318 (38.9) | 29 (42.0) |
| **Arrhythmology** | 356 (22.6) | 83 (25.2) | 244 (29.8) | 33 (47.8) |
| **Vasculitis** | 7 (0.4) | 0 (0.0) | 0(0.0) | 0(0.0) |
| **Hypertension** | 853 (54.1) | 206 (62.6) | 579 (70.8) | 52 (75.4) |
| **Hepatic failure** | 6 (0.4) | 7 (2.1) | 5 (0.6) | 1 (1.4) |
| **Gastrointestinal hemorrhage** | 96 (6.1) | 60 (18.2) | 40 (4.9) | 8 (11.6) |
| **Metabolic acidosis** | 97 (6.2) | 48 (14.6) | 18 (2.2) | 6 (8.7) |
| **Severe malnutrition** | 2 (0.1) | 2 (0.6) | 3 (0.4) | 0 (0.0) |
| **Septic shock** | 21 (1.3) | 82 (24.9) | 0 (0.0) | 9 (13.0) |
| **Multiple organ failure** | 11 (0.7) | 31 (9.4) | 10 (1.2) | 20 (29.0) |
| **Blood coagulation dysfunction** | 94 (6.0) | 52 (15.8) | 92 (11.2) | 30 (43.5) |
| **Neoplastic disease** | 381 (24.2) | 60 (18.2) | 107 (13.1) | 10 (14.5) |
| **Pleural effusion** | 319 (20.2) | 90 (27.4) | 159 (19.4) | 19 (27.5) |
| **Bronchial asthma** | 3 (0.2) | 1 (0.3) | 9 (1.1) | 0 (0.0) |
| **Malignant tumor of liver** | 23 (1.5) | 6 (1.8) | 7 (0.9) | 0 (0.0) |
| **Gastric malignant tumor** | 17 (1.1) | 0 (0.0) | 4 (0.5) | 1 (1.4) |
| **Nephrotic syndrome** | 8 (0.5) | 0 (0.0) | 0 (0.0) | 1 (1.4) |
| **Plasma adsorption** | 9 (0.6) | 17 (5.2) | 0 (0.0) | 13 (18.8) |
| **Prone position ventilation** | 106 (6.7) | 77 (23.4) | 276 (33.7) | 39 (56.5) |
| **Nasal high flow oxygen therapy** | 176 (11.2) | 136 (41.3) | 3 (0.4) | 5 (7.2) |
| **Nasal catheter for oxygen** | 37 (2.3) | 44 (13.4) | 0(0.0) | 0(0.0) |
| **Mask oxygen** | 181 (11.5) | 54 (16.4) | 27 (3.3) | 5 (7.2) |
| **Inhaled hormone** | 2 (0.1) | 6 (1.8) | 0(0.0) | 0(0.0) |
| **Thymus method new** | 145 (9.2) | 114 (34.7) | 1 (0.1) | 0 (0.0) |
| **Mechanical ventilation** | 206 (13.1) | 208 (63.2) | 58 (7.1) | 49 (71.0) |
| **Chinese patent medicine** | 458 (29.0) | 102 (31.0) | 128 (15.6) | 1 (1.4) |
| **Invasive ventilation** | 87 (5.5) | 179 (54.4) | 35 (4.3) | 49 (71.0) |
| **Oxygen therapy** | 1239 (78.6) | 286 (86.9) | 775 (94.7) | 61 (88.4) |
| **Drug allergy** | 97 (6.2) | 17 (5.2) | 57 (7.0) | 5 (7.2) |
| **Anticoagulant** | 1055 (66.9) | 280 (85.1) | 0(0.0) | 0(0.0) |
| **Glucocorticoid** | 714 (45.3) | 203 (61.7) | 0(0.0) | 0(0.0) |
| **Ribavirin** | 3 (0.2) | 5 (1.5) | 0(0.0) | 0(0.0) |
| **Antipyretic and analgesic** | 513 (32.5) | 114 (34.7) | 3 (0.4) | 0 (0.0) |
| **Oseltamivir** | 36 (2.3) | 12 (3.6) | 0(0.0) | 0(0.0) |
| **Alpha-interferon** | 10 (0.6) | 0 (0.0) | 0(0.0) | 0(0.0) |
| **Quinolone antimicrobials** | 387 (24.5) | 105 (31.9) | 0(0.0) | 0(0.0) |
| **Gamma globulin (IVIG) for intravenous injection** | 80 (5.1) | 56 (17.0) | 1 (0.1) | 0 (0.0) |
| **Aspirin (oral)** | 184 (11.7) | 52 (15.8) | 0(0.0) | 0(0.0) |
| **Cyanosis** | 1 (0.1) | 0 (0.0) | 1 (0.1) | 3 (4.3) |
| **Three concave sign** | 23 (1.5) | 10 (3.0) | 0(0.0) | 0(0.0) |
| **History of infection** | 61 (3.9) | 24 (7.3) | 20 (2.4) | 3 (4.3) |
| **Vaccination history** | 271 (17.2) | 100 (30.4) | 573 (70.0) | 50 (72.5) |
| **Travel history or residence history in the community where the case was reported** | 27 (1.7) | 13 (4.0) | 1 (0.1) | 1 (1.4) |
| **Contact history of a person infected with the novel coronavirus** | 77 (4.9) | 31 (9.4) | 12 (1.5) | 0 (0.0) |
| **Cluster onset (2 or more cases of fever and/or respiratory symptoms in small areas such as homes, offices, school classes, etc.)** | 26 (1.6) | 11 (3.3) | 0(0.0) | 0(0.0) |
